# Predicting RNA-Protein Interactions Using Only Sequence Information

**DOI:** 10.1186/1471-2105-12-489

**Published:** 2011-12-22

**Authors:** Usha K Muppirala, Vasant G Honavar, Drena Dobbs

**Affiliations:** 1 Bioinformatics and Computational Biology Program, Iowa State University, Ames, Iowa, USA; 2 Department of Genetics, Development and Cell Biology, Iowa State University, Ames, Iowa, USA; 3 Department of Computer Science, Iowa State University, Ames, Iowa, USA

## Abstract

**Background:**

RNA-protein interactions (RPIs) play important roles in a wide variety of cellular processes, ranging from transcriptional and post-transcriptional regulation of gene expression to host defense against pathogens. High throughput experiments to identify RNA-protein interactions are beginning to provide valuable information about the complexity of RNA-protein interaction networks, but are expensive and time consuming. Hence, there is a need for reliable computational methods for predicting RNA-protein interactions.

**Results:**

We propose ***RPISeq***, a family of classifiers for predicting ***R***NA-***p***rotein ***i***nteractions using only ***seq***uence information. Given the sequences of an RNA and a protein as input, *RPIseq *predicts whether or not the RNA-protein pair interact. The RNA sequence is encoded as a normalized vector of its ribonucleotide 4-mer composition, and the protein sequence is encoded as a normalized vector of its 3-mer composition, based on a 7-letter reduced alphabet representation. Two variants of *RPISeq *are presented: *RPISeq-SVM*, which uses a Support Vector Machine (SVM) classifier and *RPISeq-RF*, which uses a Random Forest classifier. On two non-redundant benchmark datasets extracted from the Protein-RNA Interface Database (PRIDB), *RPISeq *achieved an AUC (Area Under the Receiver Operating Characteristic (ROC) curve) of 0.96 and 0.92. On a third dataset containing only mRNA-protein interactions, the performance of *RPISeq *was competitive with that of a published method that requires information regarding many different features (e.g., mRNA half-life, GO annotations) of the putative RNA and protein partners. In addition, *RPISeq *classifiers trained using the PRIDB data correctly predicted the majority (57-99%) of non-coding RNA-protein interactions in NPInter-derived networks from *E. coli, S. cerevisiae, D. melanogaster, M. musculus*, and *H. sapiens*.

**Conclusions:**

Our experiments with *RPISeq *demonstrate that RNA-protein interactions can be reliably predicted using only sequence-derived information. *RPISeq *offers an inexpensive method for computational construction of RNA-protein interaction networks, and should provide useful insights into the function of non-coding RNAs. *RPISeq *is freely available as a web-based server at http://pridb.gdcb.iastate.edu/RPISeq/.

## Background

Most of the essential molecular functions of cells are governed by interactions of proteins with other proteins, nucleic acids and small ligands. Computational studies of protein interaction data have helped identify protein-protein interaction PPI networks in various organisms [1,2]. Similarly, studies on DNA-protein interactions have allowed construction of transcription factor-gene regulatory networks [3,4]. In contrast, although several ribonucleoprotein (RNP) complexes have been extensively characterized (e.g., the ribosome, the spliceosome), post-transcriptional regulatory networks that are mediated by RNA-protein interactions (RPIs) are much less well studied [5-9]. In addition to their roles in controlling gene expression at the post-transcriptional level, RPIs regulate numerous fundamental biological processes, ranging from DNA replication and transcription, to pathogen resistance, to viral replication [10-13]. Recently, high-throughput experiments have provided evidence for large numbers of RNA binding proteins in cells, and are beginning to identify and characterize pairs of RNAs and proteins that participate in RPIs [14-19]. At present, however, our understanding of RNA binding proteins lags far behind our knowledge of regulatory DNA binding proteins, such as transcription factors and replication factors.

Computational studies of RNA-protein interactions have largely focused on the "interface prediction problem", i.e., the problem of identifying the amino acid residues in a protein that are likely to bind to an RNA [20-22]. Only a few studies to date have focused on the "partner prediction problem", i.e., identification of specific RNA interaction partner(s) for a known RNA binding protein, or protein binding partner(s) for non-coding RNAs (ncRNAs). Although large-scale experimental analyses of RPIs such as RNAcompete [23], RIP-Chip [24], HITS-CLIP [25], PAR-CLIP [8] are now providing valuable data about networks of RNA-protein interactions, these experiments are expensive and time-consuming. Thus, there is a compelling need for computational methods to accurately predict RPIs and to construct RNA-protein interaction networks. Given the limited number of structurally characterized RNA-protein complexes available in the PDB [26] at present (1,092 as of June 13, 2011) and the current availability of only one database of ncRNA-protein interactions (NPInter [27]), it would be especially valuable to develop sequence-based methods that can be used to identify potential RNA-protein partners in the absence of experimental structural information regarding either partner.

Machine learning offers one of the most cost-effective approaches to constructing predictive models in settings where experimentally validated training data are available. At present, however, it is unclear whether the available experimental data regarding RNA-protein interactions are sufficient for successfully training classifiers using machine learning algorithms. Against this background, this study explores machine learning approaches to train sequence-based classifiers for predicting RPIs.

## Results

As a first step towards computational construction of RPI networks, we focused on the following question: Given the sequence of an RNA-binding protein, can we predict whether it interacts with a given RNA sequence? In developing sequence-based methods to answer this question, we considered several reduced and alternative alphabet representations of the input protein and RNA sequences. Shen *et al*. [28] used a Conjoint Triad Feature (CTF) representation to successfully predict protein-protein interactions. The CTF representation essentially encodes each protein sequence using the normalized 3-gram frequency distribution extracted from a 7-letter reduced alphabet representation of the protein sequence (See *Methods *for details). A recent study [29] demonstrated the utility of the CTF representation for predicting whether a given protein is an RNA binding protein. Inspired by these studies, we chose to encode each protein sequence using the normalized *k*-gram frequency distributions extracted from the 7-letter reduced alphabet representation of the sequence. The choice of *k *= 3 yielded the best results. We also explored several alternative representations of RNA sequences and settled on encoding each RNA sequence using normalized 4-gram frequencies extracted directly from the 4-letter ribonucleotide alphabet representation of the RNA sequence.

Our choice of Random Forest (RF) and Support Vector Machine (SVM) classifiers was motivated by several studies that have successfully used them on classification tasks that are closely related to the RPI prediction [30-33]. To rigorously evaluate the performance of these methods, we generated two non-redundant benchmark datasets, RPI2241 and RPI369, from PRIDB [34], a comprehensive database of RNA-protein complexes extracted from the PDB [26]. Most of the RNA-protein pairs in RPI2241 correspond to RPIs involving rRNAs or ribosomal proteins; the rest correspond to RPIs involving other ncRNAs or mRNAs. RPI369 corresponds to RPIs extracted from non-ribosomal complexes in RPI2241. "Negative" examples of non-interacting RNA-protein pairs were generated by randomly pairing proteins with RNAs and excluding the known interacting pairs (see *Methods *for details).

### *RPISeq *classifiers can reliably predict RNA-protein interactions

We compared the performance of *RPISeq-SVM *and *RPISeq-RF *classifiers to predict RPIs, using the benchmark datasets described above. Table 1 summarizes the prediction results obtained in 10-fold cross-validation experiments. On the RPI2241 dataset, the prediction accuracy was 89.6% (RF) and 87.1% (SVM); precision and recall for both classifiers was greater than 87%. On the RPI369 dataset, performance of both classifiers was considerably lower with an average accuracy of only 76.2% (RF) and 72.8% (SVM). Notably, values of the F-measure (weighted average of precision and recall) were greater than 0.70 for both classifiers on both datasets. Thus, the performance of classifiers estimated using 10-fold cross-validation on the larger RPI2241 dataset, which includes ribosomal data, is considerably better than that estimated using the RPI369 dataset, from which ribosomal data have been excluded. We also performed leave-one-out cross validation for the RF classifier. The results were not significantly different from 10-fold cross-validation experiments.

**Table 1:** Performance evaluation of *RPISeq*

Dataset	Classifier	Accuracy %	Precision	Recall	F-measure
RPI2241	Random Forest	89.6	0.89	0.90	0.90

RPI2241	SVM	87.1	0.87	0.88	0.87

RPI369	Random Forest	76.2	0.75	0.78	0.77

RPI369	SVM	72.8	0.73	0.73	0.73

Results of 10-fold cross-validation experiments using RPI2241 and RPI369 datasets.

See *Methods *for definitions of performance measures.

**Figure 1:**
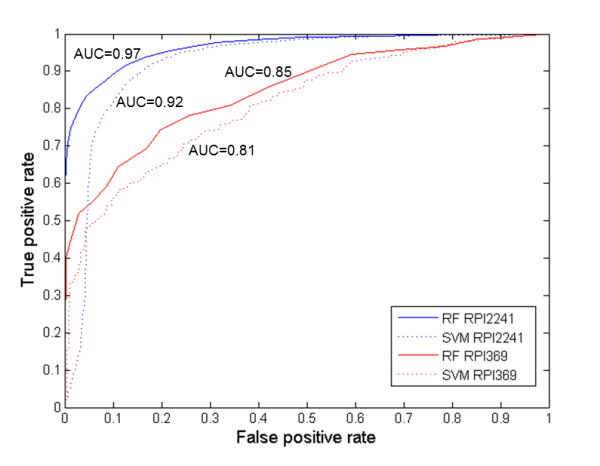
**Performance of *RPISeq *classifiers in predicting RPIs**. Receiver operating characteristic (ROC) curves for RPI predictions, illustrating the trade-off between true positive rate and false positive rate for *RPISeq-RF *(random forest) and *RPISeq-SVM *(support vector machine) classifiers, using two datasets, RPI2241 and RPI369. The area under the curve (AUC) of each ROC is shown next to the curve. The AUC for a perfect classifier is 1, and for a random classifier = 0.5.

The performance statistics reported in Table 1 were obtained using classifiers designed to provide high prediction accuracy. By varying the classification threshold value, the prediction specificity can be increased at the expense of a decrease in sensitivity. The corresponding trade-off between true positive rate and false positive rate can be seen from the receiver operating characteristic (ROC) curve shown in Figure 1. Consistent with the results in Table 1, ROC AUCs of 0.97 (RF) and 0.92 (SVM) were obtained for predictions on the RPI2241 dataset, with lower values of 0.85 (RF) and 0.81 (SVM) on the RPI369 dataset. For both classifiers, the AUC of ROC is significantly greater than 0.50 (random), indicating the feasibility of predicting RPIs using only sequence information from the RNA and protein as input.

### Comparison with other methods for predicting RNA-protein interactions

Bellucci *et al*. [35] used a variety of physicochemical properties (e.g., hydrogen-bonding propensities, secondary structure propensities) of proteins and RNAs to predict the interaction propensities for individual residues in the RNA and protein sequences of a potentially interacting pair. Because the catRAPID server [http://tartaglialab.crg.cat] does not directly report predictions as to whether or not a specific RNA-protein pair is expected to interact (the "partner prediction problem"), we were not able to directly compare our results with their method [35].

Pancaldi and Bähler *et al*. [36] also employed RF and SVM classifiers, but their method uses more than 100 different features of mRNA and proteins, extracted from the literature or computed from the protein and RNA sequences to make predictions. Examples of such features include mRNA half-life, predicted protein secondary structure, Gene Ontology annotation, relative abundance of each amino acid, codon bias. Using a dataset of 5,166 positive mRNA-protein RPI partners derived from Hogan *et al*. [10], and 5,166 randomly generated negative examples of mRNA-protein pairs, Pancaldi and Bähler reported an average accuracy of 70% in 2-fold cross-validation tests using an RF classifier based on 500 trees, and 68% using an SVM classifier using an RBF kernel with optimized parameters [36]. They also reported that 5-fold and leave-one-out experiments gave comparable results. We performed 10-fold cross-validation experiments on the same dataset using *RPISeq-RF*, which uses only sequence information. Our RF classifier achieved an accuracy of 68%, based on 500 trees, results comparable to the 70% reported for the RF classifier of Pancaldi and Bähler [36]. Our SVM classifier, using a normalized polykernel, gave less accurate predictions (61%) than the SVM of Pancaldi and Bähler (68%) [36].

In the Pancaldi and Bähler study, only 5,166 out of a total of 13,243 positive mRNA-protein pairs were actually used for prediction, because some of the features required by the classifiers were not available for the remaining 8,000 pairs [36]. When we tested our method using all 13,243 pairs for cross-validation, the prediction accuracies increased to 78% for the RF and 65% for SVM classifier. Taken together, our experiments indicate that the sequence-based method proposed here and the multiple feature-based method of Pancaldi and Bähler have comparable performance in predicting mRNA-protein interactions. Further, our results suggest that sequences of mRNAs and proteins carry sufficient information to allow reasonable predictions regarding whether or not a given mRNA and protein interact. Because feature information required by the method of Pancaldi and Bähler may not be available in many cases, our proposed method complements theirs, and may be more generally applicable for predicting ncRNA-protein partners, in addition to mRNA-protein partners.

### Predicting ncRNA-protein interaction networks

An important potential application of ***RPISeq ***is computational construction of RNA-protein interaction networks. Recently, Nacher and Araki [37] used RPIs from the NPInter database [27], a database of non-coding RNA-protein interactions, to construct non-coding RNA-protein networks for several different model organisms. Their study revealed significant similarities between ncRNA-protein and transcription factor-gene regulatory networks. To explore whether *RPISeq *could be useful for constructing networks of ncRNA-protein interactions, we evaluated our method in predicting RPIs in networks derived from NPInter. Because the NPInter RPI pairs do not include any pairs derived from ribosomes, in this experiment, we also compared the performance of models trained on the RPI369 (which lacks ribosomal sequences) versus RPI2241, to evaluate the potential effect of strong ribosomal sequence bias on performance.

Tables 2 and 3 show the number of RPI pairs correctly predicted for each organism. When trained on the RPI2241 dataset (Table 2), the RF classifier correctly predicted ~ 80% (1,349 of 1,681 total interactions). The output probabilities of *RPISeq *are estimates of interaction propensities for a specific RNA-protein pair. In Tables 2 and 3, the probability threshold used for "positive" interactions was 0.50. Among the 1,349 interactions predicted by the RF classifier, only 119 were predicted with probabilities ≥ 0.80, and another 1,230 interactions were predicted with probabilities in the range 0.50-0.80. The SVM classifier generally had slightly lower performance, correctly predicting ~ 66% of the interactions.

**Table 2:** *RPISeq *predictions on NPInter dataset using RF and SVM classifiers trained on RPI2241

Organism	**Total RPI pairs**	Pairs predicted by RF (%)	**Pairs predicted by SVM (%)**
*H. sapiens*	1189	888 (74.7)	681 (57.3)

*S. cerevisiae*	254	249 (98.0)	252 (99.2)

*M. musculus*	120	98 (81.7)	85 (70.8)

*D. melanogaster*	81	80 (98.8)	72 (88.9)

*E. coli*	37	34 (91.9)	25 (67.6)

**Total**	**1681**	**1349 (80.2)**	**1115 (66.3)**

*RPISeq *predictions on interactions derived from the NPInter database for five model organisms.

**Table 3:** RPISeq predictions on NPInter dataset using RF and SVM classifiers trained on RPI369

Organism	Total RPI pairs	Pairs predicted by RF (%)	**Pairs predicted by SVM (%)**
*H. sapiens*	1189	808 (68.0)	988 (83.1)

*S. cerevisiae*	254	168 (66.1)	226 (89.0)

*M. musculus*	120	81 (67.5)	111 (92.5)

*D. melanogaster*	81	38 (46.9)	53 (65.4)

*E. coli*	37	20 (54.0)	24 (64.9)

**Total**	**1681**	**1115 (66.3)**	**1402 (83.4)**

*RPISeq *predictions on interactions derived from the NPInter database for five model organisms.

In contrast, when trained on the RPI369 dataset, the SVM classifiers out-performed the RF classifiers (Table 3). Overall, the SVM classifier correctly predicted 1,402 (83%) and the RF classifier correctly predicted 1,115 (66%) of the interactions. Among the 1,402 interactions correctly predicted by SVM classifier, more than 850 interactions were predicted with probabilities ≥ 0.80, and another 525 interactions were predicted with probabilities in the range 0.50 to 0.80. For the RF classifier, only 50 interactions were predicted with probabilities ≥ 0.80.

With regard to the effects of ribosomal sequence bias, these results are somewhat difficult to interpret. The best "overall" prediction performance was obtained using the SVM classifier trained on the RPI369 dataset (which lacks ribosomal sequences), with 83.4% interactions correctly predicted; the RF classifier trained on the RPI2241 dataset (which includes ribosomal sequences) correctly predicted 80.2% of the total interactions. Differences in performance of classifiers trained on the two different datasets are significant when predictions for each model organism are considered individually. For example, for *D. melanogaster*, substantially better predictions were obtained with an RF classifier trained on the RPI2241 dataset (98.8%) versus an RF classifier trained on the RPI369 dataset (46.9%). In contrast, for predicting human and mouse RNA-protein interactions, SVM classifiers trained on the RPI369 dataset (which excludes the ribosomal sequences) provide the best prediction performance. For yeast RPIs, both the RF and SVM classifiers trained on RPI2241 generated excellent predictions, 98.0% and 99.2%, respectively, whereas classifiers trained on RPI369 made more errors, with correct predictions for 66.1% (RF) and 89.0% (SVM) of the cases.

**Figure 2:**
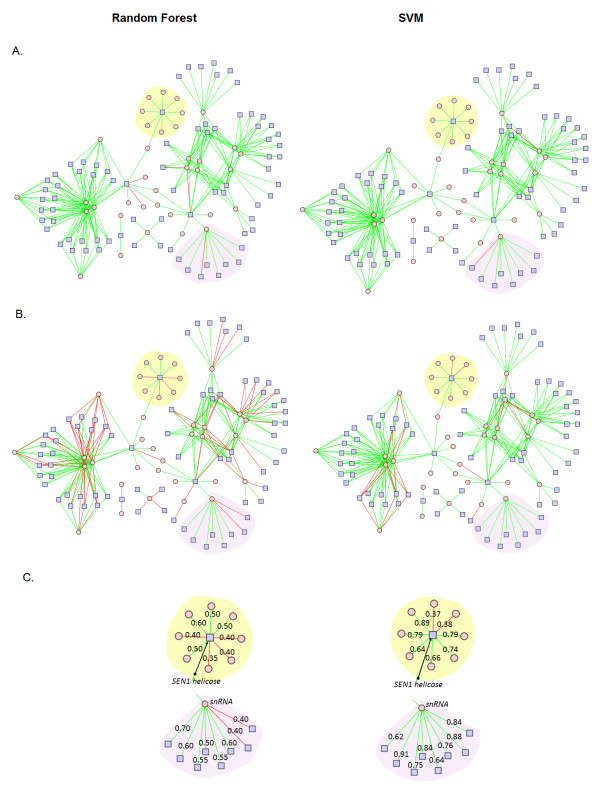
**A. Predicted interactions using classifiers trained on the RPI2241 dataset**. Among 254 known interactions, *RPISeq-RF *and *RPISeq-SVM *classifiers correctly predicted all except 5 and 2 edges, respectively. A protein hub, highlighted in yellow, shows interactions of a helicase (SEN1) with several snoRNAs. One of several RNA hubs, highlighted in purple, illustrates interactions of an snRNA (u4560) with various Sm-like proteins in the LSM complex. **B. Predicted interactions using classifiers trained on RPI369 dataset**. Among 254 known interactions, *RPISeq-RF *classifier correctly predicted 168 (66%) and *RPISeq-SVM *correctly predicted 226 (89%). A protein hub highlighted in yellow, shows interactions of a helicase (SEN1) with 8 snoRNAs. One of several RNA hubs, highlighted in purple, illustrates interactions of an snRNA (u4560) with various Sm-like proteins in the LSM complex. **C. An enlarged view of the protein (SEN1) and RNA (snRNA) hubs described in B**. above. Edges are labelled with the interaction probabilities predicted by *RPISeq-RF *(left) and *RPISeq-SVM *(right) classifiers, providing estimates of the relative pairwise interaction propensities.

Figure 2 shows the ncRNA-protein interaction network from *S. cerevisiae*, based on the data in NPInter. In Figure 2A, R*PISeq *predictions obtained using classifiers trained on the RPI2241 dataset are mapped onto the network. As described above, the SVM classifier (right) makes more correct predictions (green edges) and fewer incorrect predictions, i.e., false negatives, (red edges) than the RF classifier (left). In Figure 2B, RPI*Seq *predictions made using classifiers trained on the RPI369 dataset, which results in more errors, are shown.

One protein hub (highlighted in yellow), which appears as a green square node with connections to several RNA nodes (pink circles), is apparent in these views of the network. It corresponds to the yeast SEN-1 helicase, which is known to interact with several snoRNAs [38]. Several RNA hubs, represented by red circular nodes, each connected to several green protein nodes, are also apparent. One of these RNA hubs (highlighted in purple), corresponds to snRNA u4560, which interacts with various Sm-like proteins in the LSM complex [39].

Figure 2C shows an enlarged view of these hubs, extracted from Figure 2B. Edges are labelled with the interaction probabilities predicted by each classifier. Using classifiers trained on the RPI369 dataset, the RF classifier made more errors (i.e., predicted a known interaction with probability < 0.5) than the SVM classifier in both cases: for SEN-1 helicase, the RF classifier correctly identified only 4 out of 8 known snoRNA interactions, whereas the SVM classifier correctly identified 6 out of 8. Similarly, of 8 proteins known to interact with snRNA u4560 in yeast, the RF classifier identified 6, while the SVM classifier correctly identified all 8 interaction partners. Notably, as shown in Figure 2A, both RF and SVM classifiers trained on the RPI2241 dataset correctly identified all 8 RNA interaction partners of the SEN-1 helicase, and both classifiers missed only 1 of 8 protein interaction partners of the snRNA u4560.

## Discussion

Regulation of gene expression at the post-transcriptional level is often mediated by interactions between RNA binding proteins and mRNAs or ncRNAs [5,11,40]. In this work, we present a new method, *RPISeq*, for predicting RNA-protein interaction partners, using only sequence information, with up to 90% average accuracy. We also demonstrate, that *RPISeq *can effectively predict RNA-protein interaction networks, based on evaluation using available data from five model organisms.

### Sequence-based prediction of RNA-protein interactions

While several computational methods for predicting networks of protein-protein interactions have been developed [1,2], very few studies have focused on computational analysis or prediction of RNA-protein interactions [3,4]. One of the major challenges in solving the "partner prediction problem" for RNA-protein interactions is the limited amount of experimental data currently available. Unlike the "interface prediction problem," for which detailed structural information for more than 1,000 RNA-protein complexes is available in the PDB, mRNA partners for only a handful of RBPs are known [10]. Currently, two basic types of information regarding RNA-protein interaction partners are widely available: i) experimentally-determined structures of RNA-protein complexes, available in primary resources such as the PDB [26] and NDB [41], and secondary resources such as PRIDB [34] and BIPA [42]; and ii) experimental data from *in vivo *or *in vitro *cross-linking studies focused on individual proteins (e.g., SFRS1 [43], PUF [44]) or from high throughput RNA-binding microarrays [23], stored in repositories such as NPInter [27], CLIPZ [45] and RBPDB [46].

*RPISeq *requires only sequence information to generate predictions. In the current version of *RPISeq*, the classifiers were trained using only RPIs for which experimental structures are available. RPI2241 is a non-redundant training dataset consisting of 2241 interacting RNA-protein pairs, and includes a wide variety of different functional classes of proteins and RNA (e.g., rRNA, tRNA, miRNA, mRNA). rRNA-ribosomal protein pairs constitute ~ 40% of the total, reflecting the predominance of ribosomal structures in the current version of the PDB. To investigate the impact of this bias on machine learning methods for predicting RPIs, we also generated a smaller dataset of 369 RNA-protein partners (RPI369), from which all rRNA-containing complexes had been removed (see *Methods *for details).

We used RPI2241 and RPI369 as non-redundant benchmark datasets for developing and rigorously evaluating the performance of various machine learning classifiers. In cross-validation experiments, classifiers trained and tested on the larger dataset had superior prediction performance, indicating that the greater number and diversity of complexes in RPI2241, relative to RPI369, has a stronger positive effect on classification accuracy than the potentially negative effect of sequence bias in RPI2241. When we evaluated classifiers using independent datasets of RPIs from NPInter, however, classifiers trained on RPI369, in some cases, had better prediction performance. The basis for this observation is currently under investigation.

To identify sequence features of the proteins and RNA important in determining their specific interactions, we analyzed the features most frequently used by the Random Forest classifier to predict interacting partners (see *Methods *for details).

The four most often selected RNA tetrads were: *AUUC, AGUG, UUUU and UCAA*. Notably, these tetrads were found in the interfacial region in only 15% of the cases examined. The most frequently selected conjoint triad in protein sequences was {*I, L, F, P*}{*A, G, V*}{*R, K*}, which represents twenty-four possible amino acid triplets (e.g., *IAR, IAK, IGR, IGK*...). The complete list of important RNA and protein features is provided as Supplemental data S1 (Additional file 1). Although additional experiments and analyses of these features will be required to extract precise "rules" that specify a particular RNA-protein interaction, our current analysis indicates that at least 50 features (a combination of RNA and protein features) are required to accurately classify a given RNA-protein pair as interacting or not.

In this study, *RPISeq *accurately predicted RPIs in both cross-validation experiments using the benchmark datasets and in experiments on independent datasets. This suggests that normalized *k*-mer frequency distributions of RNA and protein sequences (specifically, reduced alphabet representations of protein sequences) in combination with appropriate machine learning methods, provide an effective approach to construct RPI predictors. Because the data used in this study represent only a small fraction of cellular RNA-protein complexes and interactions, we anticipate that more accurate predictions will be possible when larger and more diverse datasets of experimentally validated RPIs become available.

### Comparison with other available methods

The method of Pancaldi and Bähler [36], which was developed to predict mRNA-protein interactions (rather than ncRNA-protein interactions), also uses RF and SVM classifiers, but requires a much more extensive set of features regarding the mRNAs and proteins. Input for the classifiers, which consists of a vector constructed by concatenating the features of potential RNA and protein partners (e.g., isoelectric point of protein, protein localization, mRNA half-life), cannot be extracted or calculated from sequence information alone. This requirement restricts the applicability of this method in practice: Pancaldi and Bähler were not able to extract the necessary features for a majority of interactions in their RPI dataset. The *RPISeq *methods do not suffer from this limitation because they require only sequence-derived features to make reliable predictions. In fact, the performance of *RPISeq *improved substantially (by 8% in accuracy) when evaluated on the entire dataset of Pancaldi and Bähler. Thus, for predicting mRNA-protein interactions, the sequence-based approach implemented in *RPISeq *provides performance comparable to that of classifiers that require a more extensive set of features, including those that cannot be extracted from RNA and protein sequences alone.

### Application of *RPISeq *to constructing RNA-protein interaction networks

Encouraged by the success of *RPISeq *in predicting specific RPIs, we examined its effectiveness in constructing RNA-protein interaction networks in several model organisms, using only information derived from RNA and protein sequences. The networks were extracted from the "ncRNA binds protein" category of NPInter [27], currently the only available database of functional interactions of ncRNA with proteins. *RPISeq *was able to successfully predict the interactions of a single protein with multiple RNAs (protein hubs), as well as interactions of a single RNA with multiple proteins (RNA hubs).

In the case of the yeast, *S. cerevisiae*, *RPISeq *provided excellent predictions of RPIs: both the RF and SVM classifiers trained on the RPI2241 dataset correctly predicted > 98% of interactions in the NPInter database [27]. The *RPISeq-RF *classifier trained on the RPI2241 dataset also correctly identified a large majority of interactions in the *D. melanogaster *(99%) and *E. coli *(92%) networks. For human and mouse networks, however, classifiers trained on the RPI369 dataset gave better performance, with the *RPISeq-SVM *classifier correctly identifying 83% of the interactions in human and 93% in the mouse. It is important to note that these evaluations are based on predicting only known *"positive" *interactions currently available in NPInter [27]; *"negative" *data regarding non-interacting protein-RNA-protein pairs are not included in NPInter. Because the experimental data in NPInter are incomplete, it is problematic to assume that RNA-protein pairs not included in NPInter do not, in fact, interact. Also, some experimentally-determined RPIs included in NPInter could correspond to false positives.

Given the relatively small sizes of the RNA-protein networks analyzed in this study, differences in the results obtained using different classifiers to predict RPIs in different species must be interpreted with caution. It will be important to evaluate these methods on larger, more complete datasets of experimentally validated RNA-protein interactions as they become available. On the whole, our results suggest that *RPISeq *should be valuable for constructing and analyzing regulatory RNA-protein interaction networks.

## Conclusion

In this work, we tested whether *RPISeq*, a family of purely sequence-based classifiers, can be used to predict whether a specific RNA-protein pair is likely to interact. Our results demonstrate that the corresponding RNA and protein sequences alone contain sufficient information to allow reliable prediction of RPIs. Such predictions can be used to: (i) identify putative RNA partners of a target protein, or protein partners of a target RNA; and (ii) computationally construct RNA-protein interaction networks. The datasets used in this study are relatively small compared with the large number of RNA-protein complexes and diverse interactions that occur in cells. The increasing availability of transcriptome-wide experimental data should lead to improvements in computational methods for predicting RNA-protein interactions and for modelling regulatory networks of RNA-protein interactions. *RPISeq *is freely available as a web-based server at http://pridb.gdcb.iastate.edu/RPISeq/.

## Methods

### RPI benchmark datasets derived from structure-based experimental data

For training and testing classifiers, two benchmark non-redundant datasets of RNA-protein interacting pairs were extracted from 943 protein-RNA complexes in PRIDB using an 8 Å distance cut-off [34]. PRIDB is a database of protein-RNA interfaces calculated from protein-RNA complexes in the PDB [26]. The original 943 complexes from PRIDB contained a total of 9,689 protein chains and 2,074 RNA chains; the final dataset RPI2241 (see below), which contains a total of 952 protein chains and 443 RNA chains, was derived from these complexes by applying the following criteria. Redundant protein sequences (i.e., with ≥ 30% sequence identity) interacting with similar RNA sequences (i.e., with ≥ 30% sequence identity) were discarded. Also, redundant RNA sequences (i.e., with ≥ 30% sequence identity) interacting with similar protein sequences (i.e., with ≥ 30% sequence identity) were discarded. Only proteins whose length is greater than 25 and RNAs at least 15 nucleotides long were retained. This resulted in a dataset of "positive" examples, RPI2241, consisting of 2241 experimentally validated RNA-protein pairs, and is provided as Supplemental data S2 (Additional file 2).

To generate a balanced dataset of "non-interacting RNA-protein pairs" (negative examples), we randomly paired the RNAs and proteins from the 943 protein-RNA complexes and removed similar interacting RNA-protein pairs (a randomly generated pair A-B was discarded if there exists a positive interaction pair C-B, and A and C share ≥ 30% sequence identity). Because ~40% of RNA-protein complexes in the PDB correspond to ribosomal structures, the RPI2241 dataset is also strongly biased towards ribosomal RPIs. Thus, we constructed a second dataset, RPI369, which is a subset of RPI2241 generated by removing all RPIs that contain ribosomal proteins or ribosomal RNAs and is provided as Supplemental data S3 (Additional file 3). RPI369 contains only non-ribosomal complexes (e.g., tRNA, mRNA, viral RNA, miRNA).

### RPI datasets derived from non-structure-based experimental data

For evaluation of our method on independent RPI datasets, we used two datasets of RPIs obtained from RNA immunoaffinity purification and microarray experiments, published by Hogan *et al *[10]. One dataset comprises 5,166 mRNA-protein interactions; this dataset was also used in the study of Pancaldi and Bähler [36]. The second dataset is larger, consisting of 13,243 RPIs, and including all 5,166 interactions in the smaller dataset. Pancaldi and Bähler were not able to evaluate their method on this larger dataset because of missing feature information for RNAs and proteins involved in these interactions. Because *RPISeq *uses only sequence information, we were able to evaluate our method using all of the available data.

To test the ability of *RPISeq to *predict ncRNA-protein interaction networks, we used the NPInter database http://www.panrna.org/NPInter/, which includes eight different categories of functional interactions between non-coding RNAs, but excludes ribosomal RNAs and proteins. We extracted only those interactions for which there is experimental evidence for physical association of ncRNA with a protein, i.e. the 'ncRNA binds protein' category.

### Alternative representations of protein and RNA sequences

Each RNA-protein pair is represented as a 599-feature vector, in which 343 features are used to encode the protein sequence and 256 features are used to encode the RNA sequence. Proteins are encoded using the conjoint triad feature (CTF) representation previously used by Shen *et al *[28]. In this method, the 20 amino acids are classified into 7 groups according to their dipole moments and the volume of their side chains: {*A*, *G*, *V*}, {*I*, *L*, *F*, *P*}, {*Y*, *M*, *T*, *S*}, {*H*, *N*, *Q*, *W*}, {*R*, *K*}, {*D*, *E*}, {*C*}. Each protein sequence is then encoded using the 7-letter reduced alphabet. Each protein feature represents the normalized frequency of the corresponding conjoint triad, i.e., 3-mer in the 7-letter reduced alphabet representation of the protein sequence. Thus, each protein sequence is represented by a 343 (7 × 7 × 7) dimensional vector, where each element of the vector corresponds to the normalized frequency of the corresponding 3-mer in the sequence (see [28] for details). Based on results of preliminary tests comparing the normalized *k*-mer frequency representation of RNA sequences for different values of *k*, we chose to encode RNA sequences using a 4 × 4 × 4 or 256-dimensional vector, in which each feature represents the normalized frequency of the corresponding 4-mer appearing in the RNA sequence (e.g., *AAUG, CGAU, GGCC*)

### Machine Learning Algorithms

The SVM classifier [47] classifies input samples represented in the form of n-dimensional vectors into two classes using a hyperplane in a feature space. If the patterns are not separable in the original *n*-dimensional input space, a suitable non-linear kernel function is used to implicitly map the patterns in the *n*-dimensional input space into a typically higher (finite or even infinite) dimensional kernel-induced feature space in which the patterns become separable or nearly separable. Given a training set consisting labeled examples of the form (X_i_, y_i_) where X_i _is an n-dimensional input vector and y_i _= 0/1 is its label (i.e., the desired output of the SVM classifier for input X_i_), the SVM learning algorithm effectively selects the hyperplane that maximizes the margin of separation between the training samples of the two classes from among all separating hyperplanes. If the examples are not perfectly separable in the kernel-induced feature space, a user-chosen parameter C is used to trade off training error (the number of misclassified training examples) against margin for the correctly classified training examples.

In our study, the input to the SVM classifier is a 599-dimensional vector that encodes features of a given pair of RNA and protein sequences as described above. The output of the SVM is a binary label indicating whether the given RNA-protein pair interact or not. We used the Sequential Minimal Optimization SMO implementation in Weka 3.7 [48] to train the SVM classifier. After some preliminary experiments which showed that the normalized polykernel performed better than RBF kernel on our data, we chose the normalized polykernel function of order 2 with *ε *= 1.0E-12. We set C = 1.0 and tolerance parameter T = 0.0010. We then used the option to fit a logistic model to the output of the resulting SVM classifier to obtain the posterior probability of class from the SVM output for any given input.

RandomForest (RF) [49] is an ensemble of many classification trees. Each tree in the ensemble is trained on a subset of training examples that are randomly sampled from the given training set. At each node the best split is chosen from a set of *m *variables selected at random from the set of input features. Given a query instance, the majority vote of all the classifiers is returned as the RF prediction. We used the Random Forest implementation in Weka 3.7. By default, Weka builds a RF classifier as an ensemble of 10 trees and sets the value of *m *= log_2 (number of features) + 1. For most of our experiments, we set the number of trees to 20 and 10 features were evaluated at each node. For comparison with the method of Pancaldi and Bähler [36], we set the number of trees to 500.

For performing feature selection, we used *AttributeSelection *class in Weka toolkit. We used *wrapper subset evaluator *in combination with Random Forest classifier and best first search method.

### Performance Evaluation

Standard 10-fold cross-validation procedures were used to evaluate and compare classifier performance on the benchmark datasets. For the RF classifier, we also performed leave-one-out cross-validation; results were not significantly different from those obtained using 10-fold cross-validation (data not shown).

We computed the following statistics, as described in Baldi *et al*. [50], to measure the performance of the classifiers.

$$\begin{array}{c} precision=\frac{TP}{TP+FP}\\ Recall=\frac{TP}{TP+FN}\\ Accuracy=\frac{TP+TN}{TP+TN+FP+FN}\\ F\text{ - }Measure=\frac{2\times Precision\times Recall}{Precision+Recall} \end{array}$$

where TP is the number of true positives, FP is the number of false positives, TN is the number of true negatives, and FN is the number of false negatives.

The F-Measure is a composite indicator of performance that attempts to "balance" precision and recall. F-Measure values range from 0 to 1, with values close to 1 indicating better performance. The area under the curve (AUC) of the receiver operating characteristic curve (ROC) was also computed. AUC values also range from 0 to 1: the AUC = 1 for a perfect classifier and for a random classifier = 0.5.

## Authors' contributions

UKM conceived the study (with DD and VGH), carried out the experiments, implemented the *RPISeq *webserver and prepared the initial draft of the manuscript. DD and VGH contributed to the experimental design, supervised the work, and edited the manuscript. All authors read and approved the final manuscript.

## Supplementary Material

Additional file 1 **List of RNA and protein features important for distinguishing interacting and non-interacting RNA-protein pairs (S1)**. Click here for file

Additional file 2 **Positive RPIs in the RPI2241 dataset**. This is a tab-delimited file with two columns. The first column is a list of proteins and the second column is a list of corresponding RNAs (S2). Click here for file

Additional file 3 **Positive RPIs in RPI369 dataset**. This is a tab-delimited file with two columns. The first column is a list of proteins and the second column is a list of corresponding RNAs (S3). Click here for file

## References

[B1] Lees JG Heriche JK Morilla I Ranea JA Orengo CA Systematic computational prediction of protein interaction networks Phys Biol 2011 8 035008 10.1088/1478-3975/8/3/035008 21572181

[B2] Wang T-Y He F Hu Q-W Zhang Z A predicted protein-protein interaction network of the filamentous fungus Neurospora crassa Mol Biosyst 2011 10.1039/c1mb05028a 21584303

[B3] Lee TI Transcriptional regulatory networks in Saccharomyces cerevisiae Science 2002 298 799 804 10.1126/science.1075090 12399584

[B4] Martínez-antonio A Escherichia coli transcriptional regulatory network Netw Biol 2011 1 21 33 10.1016/j.jmb.2008.05.054 PMC2726282 18599074

[B5] Kishore S Luber S Zavolan M Deciphering the role of RNA-binding proteins in the post-transcriptional control of gene expression Brief Funct Genomics 2010 9 391 404 10.1093/bfgp/elq028 21127008 PMC3080770

[B6] Mittal N Roy N Babu MM Janga SC Dissecting the expression dynamics of RNA-binding proteins in posttranscriptional regulatory networks Proc Natl Acad Sci USA 2009 106 20300 20305 10.1073/pnas.0906940106 19918083 PMC2777960

[B7] Tsvetanova NG Klass DM Salzman J Brown PO Proteome-wide search reveals unexpected RNA-binding proteins in Saccharomyces cerevisiae PLoS One 2010 5 e12671 10.1371/journal.pone.0012671 20844764 PMC2937035

[B8] Hafner M Landthaler M Burger L Khorshid M Hausser J Berninger P Rothballer A Ascano M Jr Jungkamp AC Munschauer M Ulrich A Wardle GS Dewell S Zavolan M Tuschl T Transcriptome-wide identification of RNA-binding protein and microRNA target sites by PAR-CLIP Cell 2010 141 129 141 10.1016/j.cell.2010.03.009 20371350 PMC2861495

[B9] Hafner M Landthaler M Burger L Khorshid M Hausser J Berninger P Rothballer A Ascano M Jr Jungkamp AC Munschauer M Ulrich A Wardle GS Dewell S Zavolan M Tuschl T PAR-CliP--a method to identify transcriptome-wide the binding sites of RNA binding proteins J Vis Exp 2010 10.3791/2034 PMC3156069 20644507

[B10] Hogan DJ Riordan DP Gerber AP Herschlag D Brown PO Diverse RNA-binding proteins interact with functionally related sets of RNAs, suggesting an extensive regulatory system PLoS Biol 2008 6 e255 10.1371/journal.pbio.0060255 18959479 PMC2573929

[B11] Licatalosi DD Darnell RB RNA processing and its regulation: global insights into biological networks Nat Rev Genet 2010 11 75 87 20019688 10.1038/nrg2673 PMC3229837

[B12] Sola I Mateos-Gomez PA Almazan F Zuñiga S Enjuanes L RNA-RNA and RNA-protein interactions in coronavirus replication and transcription RNA Biol 2011 8 237 248 10.4161/rna.8.2.14991 21378501 PMC3230552

[B13] Li Z Nagy PD Diverse roles of host RNA binding proteins in RNA virus replication RNA Biol 2011 8 305 315 10.4161/rna.8.2.15391 21505273 PMC3230553

[B14] Baroni TE Chittur SV George AD Tenenbaum SA Advances in RIP-chip analysis: RNA-binding protein immunoprecipitation-microarray profiling Methods Mol Biol 2008 419 93 108 10.1007/978-1-59745-033-1_6 18369977

[B15] Barkan A Genome-wide analysis of RNA-protein interactions in plants Methods Mol Biol 2009 553 13 37 10.1007/978-1-60327-563-7_2 19588099

[B16] Charon C Moreno AB Bardou F Crespi M Non-protein-coding RNAs and their interacting RNA-binding proteins in the plant cell nucleus Mol Plant 2010 3 729 739 10.1093/mp/ssq037 20603381

[B17] Kaymak E Wee LM Ryder SP Structure and function of nematode RNA-binding proteins Curr Opin Struct Biol 2010 20 305 312 10.1016/j.sbi.2010.03.010 20418095 PMC2916969

[B18] Kim MY Hur J Jeong S Emerging roles of RNA and RNA-binding protein network in cancer cells BMB Rep 2009 42 125 130 10.5483/BMBRep.2009.42.3.125 19335997

[B19] Pacheco A Martinez-Salas E Insights into the biology of IRES elements through riboproteomic approaches J Biomed Biotechnol 2010 doi:10.1155/2010/458927 10.1155/2010/458927 PMC2817807 20150968

[B20] Terribilini M Lee J-H Yan C Jerniga RL Honavar V Dobbs D Prediction of RNA binding sites in proteins from amino acid sequence RNA 2006 12 1450 62 10.1261/rna.2197306 16790841 PMC1524891

[B21] Pérez-Cano L Fernández-Recio J Optimal protein-RNA area, OPRA: a propensity-based method to identify RNA-binding sites on proteins Proteins 2010 78 25 35 10.1002/prot.22527 19714772

[B22] Zhou P Zou J Tian F Shang Z Geometric similarity between protein-RNA interfaces J Comput Chem 2009 30 2738 2751 10.1002/jcc.21300 19399760

[B23] Ray D Kazan H Chan ET Castillo LP Chaudhry S Talukder S Blencowe BJ Morris Q Hughes TR Rapid and systematic analysis of the RNA recognition specificities of RNA-binding proteins Nature Biotechnol 2009 27 667 70 10.1038/nbt.1550 19561594

[B24] Keene JD Komisarow JM Friedersdorf MB RIP-Chip: the isolation and identification of mRNAs, microRNAs and protein components of ribonucleoprotein complexes from cell extracts Nature protoc 2006 1 302 7 10.1038/nprot.2006.47 17406249

[B25] Licatalosi DD Mele A Fak JJ Ule J Kayikci M Chi SW Clark TA Blume JE Wang X Darnell JC Darnell RB HITS-CLIP yields genome-wide insights into brain alternative RNA processing Nature 2008 456 464 9 10.1038/nature07488 18978773 PMC2597294

[B26] Berman HM Westbrook J Feng Z Gilliland G Bhat TN Weissig H Shindyalov IN Bourne PE The Protein Data Bank Nucleic Acids Res 2000 28 235 42 10.1093/nar/28.1.235 10592235 PMC102472

[B27] Wu T Wang J Liu C Zhang Y Shi B Zhu X Zhang Z Skogerbø G chen L Lu H Zhao Y Chen R NPInter: the noncoding RNAs and protein related biomacromolecules interaction database Nucleic Acids Res 2006 34 D150 2 10.1093/nar/gkj025 16381834 PMC1347388

[B28] Shen J Zhang J Luo X Zhu W Yu K Chen K Li Y Jiang H Predicting protein-protein interactions based only on sequences information Proc Natl Acad Sci USA 2007 104 4337 41 10.1073/pnas.0607879104 17360525 PMC1838603

[B29] Shao X Tian Y Wu L Wang Y Jing L Deng N Predicting DNA-and RNA-binding proteins from sequences with kernel methods J Theor Biol 2009 258 289 293 10.1016/j.jtbi.2009.01.024 19490865

[B30] Wang Y Wang J Yang Z Deng N Sequence-based protein-protein interaction prediction via support vector machine J Syst Sci Complex 2010 23 1012 1023 10.1007/s11424-010-0214-z

[B31] Hwang H Vreven T Whitfield TW Wiehe K Weng Z A machine learning approach for the prediction of protein surface loop flexibility Proteins: Struct Funct Bioinf 2011 79 doi: 10.1002/prot.23070 10.1002/prot.23070 PMC3341935 21633973

[B32] Chen X-W Liu M Prediction of protein-protein interactions using random decision forest framework Bioinformatics 2005 21 4394 400 10.1093/bioinformatics/bti721 16234318

[B33] Liu Z-P Wu L-Y Wang Y Zhang X-S Chen L Prediction of protein-RNA binding sites by a random forest method with combined features Bioinformatics 2010 26 1616 1622 10.1093/bioinformatics/btq253 20483814

[B34] Lewis BA Walia RR Terribilini M Feguson J Zheng C Honavar V Dobbs D PRIDB: a Protein-RNA Interface Database Nucleic Acids Res 2011 39 D277 82 10.1093/nar/gkq1108 21071426 PMC3013700

[B35] Bellucci M Agostini F Masin M Tartaglia GG Predicting protein associations with long noncoding RNAs Nature Methods 2011 8 444 445 10.1038/nmeth.1611 21623348

[B36] Pancaldi V Bähler J In silico characterization and prediction of global protein-mRNA interactions in yeast Nucleic Acids Res 2011 1 11 10.1093/nar/gkr160 PMC3152324 21459850

[B37] Nacher JC Araki N Structural characterization and modeling of ncRNA-protein interactions Biosystems 2010 101 10 9 10.1016/j.biosystems.2010.02.005 20206662

[B38] Ursic D Chinchilla KJSF Culbertson MR Multiple protein/protein and protein/RNA interactions suggest roles for yeast DNA/RNA helicase Sen1p in transcription, transcription-coupled DNA Nucleic Acids Res 2004 32 2441 2452 10.1093/nar/gkh561 15121901 PMC419450

[B39] Vidal VP Verdone L Mayes AE Beggs JD Characterization of U6 snRNA-protein interactions RNA 1999 5 1470 81 10.1017/S1355838299991355 10580475 PMC1369868

[B40] Blencowe B Brenner S Hughes T Morris Q Post-transcriptional gene regulation: RNA-protein interactions, RNA processing, mRNA stability and localization Pac Symp Biocomput 2009 545 548 19209730

[B41] Berman HM Olson WK Beveridge DL Westbrook J Gelbin A Demeny T Hsieh S-H Srinivasan AR Schneider B A comprehensive relational database of three-dimensional structures of nucleic acids Biophys J 1992 63 751 759 10.1016/S0006-3495(92)81649-1 1384741 PMC1262208

[B42] Lee S Blundell T BIPA: a database for protein-nucleic acid interaction in 3D structures Bioinformatics 2009 25 1559 1560 10.1093/bioinformatics/btp243 19357098

[B43] Sanford JR Wang X Mort M VanDyun N Cooper DN Mooney SD Edenburg HJ Liu Y Splicing factor SFRS1 recognizes a functionally diverse landscape of RNA transcripts Genome Res 2009 19 381 94 19116412 10.1101/gr.082503.108 PMC2661799

[B44] Gerber AP Herschlag D Brown PO Extensive association of functionally and cytotopically related mRNAs with Puf family RNA-binding proteins in yeast PLoS Biol 2004 2 E79 10.1371/journal.pbio.0020079 15024427 PMC368173

[B45] Khorshid M Rodak C Zavolan M CLIPZ: a database and analysis environment for experimentally determined binding sites of RNA-binding proteins Nucleic Acids Res 2010 39 245 252 10.1093/nar/gkq940 PMC3013791 21087992

[B46] Cook KB Kazan H Zuberi K Morris Q Hughes TR RBPDB: a database of RNA-binding specificities Nucleic Acids Res 2010 39 301 308 10.1093/nar/gkq1069 PMC3013675 21036867

[B47] Vapnik V The Nature of Statistical Learning Theory 1995 New York: Springer

[B48] Hall M Frank E Holmes G Pfahringer B Reutemann P Witten IH The WEKA data mining software: An update SIGKDD Explorations 2009 11 10 18 10.1145/1656274.1656278

[B49] Breiman I Random Forests Mach Learn 2001 45 5 32 10.1023/A:1010933404324

[B50] Baldi P Brunak S Chauvin Y Andersen CAF Nielsen H Assessing the accuracy of prediction algorithms for classification: An overview Bioinformatics 2000 16 412 424 10.1093/bioinformatics/16.5.412 10871264

